# Diagnostic Challenges in Neuro-Psychiatric Systemic Lupus Erythematosus: A Case of a Patient With Psychosis Secondary to Lupus Cerebritis in the Setting of a Steroid Taper

**DOI:** 10.7759/cureus.64484

**Published:** 2024-07-13

**Authors:** Nicholas Fazio, Monica Raiss, Ronak Shah, Vaibhav Vagal, Maxwell Moore, Mason Chacko

**Affiliations:** 1 Psychiatry, Stony Brook University, Stony Brook, USA

**Keywords:** psychosis, steroid induced psychosis, cerebritis, neuropsychiatric systemic lupus erythematosus (npsle), systemic lupus erythromatosus

## Abstract

Systemic lupus erythematosus (SLE) is an autoimmune condition whereby autoantibodies target systemic tissues, causing manifestations of inflammation and tissue damage. Neurologic inflammation in SLE can cause an array of neuropsychiatric (NP) symptoms, including headaches, depression, seizures, demyelinating conditions, mania, and psychosis. Patients treated for SLE are often on anti-inflammatory regimens, including high-dose steroids, which can independently precipitate psychosis. Steroid-induced psychosis (SIP) and lupus cerebritis (LC) are two distinct conditions that patients with SLE may have but often have overlapping presentations, which present a challenge for clinicians. Accurately differentiating between SIP and LC in an emergency setting is crucial for directing appropriate management and preventing potential complications. A clear timeline of the history of symptoms can help narrow down the cause. Diagnostic tools, mainly MRI patterns, can further clarify and indicate the presence of LC. We present a case of a 19-year-old African American female with a history of one steroid-induced psychotic episode five months prior in the setting of SLE who developed subsequent psychosis while on a steroid taper. MRI imaging elucidated a diagnosis of LC rather than a second SIP episode. There are few, if any, case reports that describe a patient with past SIP with a subsequent flare of cerebritis with psychotic symptoms. Strategic approaches to differentiating SIP from LC in the setting of SLE can lead to improved patient outcomes, follow-up care, and an overall understanding of the neuropsychiatric complexities of SLE.

## Introduction

Systemic lupus erythematosus (SLE) is a complex autoimmune condition affecting multiple organ systems. Neuropsychiatric systemic lupus erythematosus (NPSLE) covers a constellation of symptoms from involvement with the central nervous system (CNS), peripheral nervous system (PNS), and psychiatric disorders. Because of the varied clinical presentations, the American College of Rheumatology (ACR) has defined 19 distinct clinical entities of NPSLE to standardize diagnosis [1]. Patients with SLE have shown up to a 56.3% prevalence of neuropsychiatric (NP) events of the 19 clinical entities, with headaches, mood disorders, cognitive dysfunction, and seizures being among the most common [2]. A key to diagnosing NPSLE is that the neurologic or psychiatric manifestations are attributed to SLE, not another cause. Identifying the etiology of psychiatric manifestations in SLE can be particularly challenging. Psychosis, specifically, is a rare and debilitating symptom seen in patients with SLE and may be due to NPSLE, side effects of SLE treatment, or an exacerbation of a premorbid psychiatric condition [3].

Lupus cerebritis (LC) falls under the category of NPSLE and specifically refers to brain inflammation associated with SLE. Prospective studies of patients with SLE have shown that CNS involvement correlates with high disease activity, and in the setting of those with any major CNS involvement, acute psychosis is rare, only contributing to 4.3% of the total CNS events [4]. Researchers have postulated that inflammation of the CNS breaks down the blood-brain barrier, causing immune cells and autoantibodies to attack brain tissue [1,5]. These autoantibodies can precipitate into immune complexes that deposit within neural tissue as well as blood vessels, furthering neuroinflammation [6]. Lupus flares are pro-inflammatory states, and high levels of cytokines can predispose patients to psychiatric symptoms by disrupting the homeostasis of neurotransmitters in the brain [5]. MRI and CSF studies are the gold standard to differentiate LC from other neurologic disturbances. CSF studies typically show white cells, protein, immunoglobulin synthesis, or absolute immunoglobulin G (IgG) [7,8]. Radiographic findings include subcortical and deep white matter hyperintensities or confluent white matter lesions [9]. Managing psychosis in the setting of SLE necessitates treating the underlying lupus flare with immunosuppressive therapies and antipsychotic medications.

The treatment of severe SLE in itself may also induce psychosis as well as mania. Oral glucocorticoids treat mild to moderate disease, whereas severe flares may warrant high-dose IV glucocorticoids. Some individuals are sensitive to their systemic effects, especially on the CNS. Since certain patients with SLE may need to take a prolonged course of high-dose steroids, they are at higher risk of steroid-induced psychosis (SIP). In such cases, discontinuing the steroids and administering antipsychotics may be necessary [3,10,11].

The varied causes of psychosis in SLE make an appropriate diagnosis and treatment imperative. However, limited data exist on how to do this optimally. We present a case study of a patient with SLE actively treated with high-dose steroids who developed psychotic symptoms. By considering the patient's history and employing appropriate diagnostic studies, clinicians can appropriately identify the etiology of psychosis and begin the appropriate treatment.

## Case presentation

Admission

A 19-year-old African American female with SLE presented to the emergency department (ED) for agitation and confusion. She was diagnosed five months prior to this presentation with SLE and was treated with steroids, but then developed psychotic symptoms during that steroid treatment. She had no personal psychiatric history prior to those initial events and no family psychiatric history. Her social history was positive for social alcohol use, denying other substance use. Her last lupus flare, without any psychotic symptoms on presentation, was one month before this admission. On presentation to our ED, the patient was finishing a course of prednisone, from 40 mg, tapered weekly by 10 mg, and currently on 20 mg. Her home medication regimen included Myfortic 720 mg twice daily, trazodone 50 mg nightly, hydroxychloroquine 400 mg daily, Lisinopril 5 mg daily, and prednisone taper 20 mg daily. She initially failed azathioprine but was previously treated with cyclophosphamide and rituximab three months prior. Days before her ED presentation, her mother stated she had intermittent aggressive behaviors and paranoid delusions regarding family members, like "hiding around the house and spying on her." En route to the ED, the patient had to be physically restrained due to aggressive behavior. Her initial mental exam in the ED was notable for drowsiness, poor eye contact, mumbling speech, a confused mood, constricted affect, poor insight, and poor impulse control. The initial differential diagnosis was broad, including steroid-induced psychosis, autoimmune encephalitis, lupus exacerbation, substance use, infectious encephalitis, and an isolated brief psychotic disorder.

Laboratory studies, brain imaging, and hospital course

**Figure 1:**
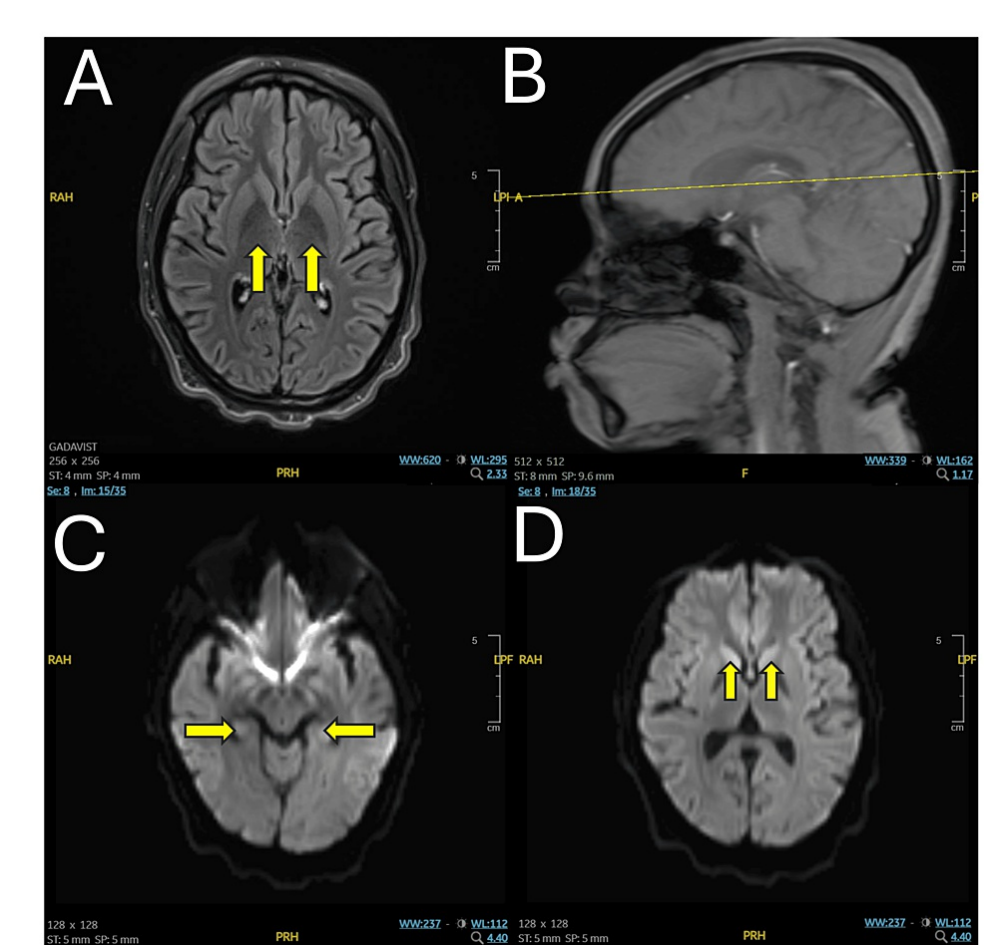
MRI brain (A) Axial view T2 flair, yellow arrows indicating posterior limb of the internal capsule, (B) sagittal view T2 flair, yellow cross-section of view from panel A, (C) axial view diffusion tensor imaging, yellow arrows indicating parahippocampal gyri, (D) axial view diffusion tensor imaging, yellow arrows indicating caudate nuclei head.

The patient's laboratory studies were notable for microcytic anemia, elevated erythrocyte sedimentation rate (ESR), positive lupus anticoagulant, and elevated C-reactive protein with a normal infectious panel and normal urinary toxicology (Table 1). On MRI, studies found subtle findings of symmetric swelling with FLAIR hyperintensity and diffusion-weighted imaging (DWI) hyperintensity/ADV hypointensity representing restricted diffusion at the bilateral posterior limb of the internal capsule, caudate nuclei head, and parahippocampal gyri (Figure 1), indicating possible encephalitis and early findings of hypoxic-ischemic encephalopathy. The hospital course is as follows in Table 2, where increasing the patient's steroid dose by 500 mg for three days markedly improved the patient's mood and mental status by day 3 and overall systemic symptoms of SLE by days 4-7. The addition of olanzapine on day 1 also showed marked improvement in mood, a return to the neuropsychiatric baseline, and a remission of symptoms of psychosis. Given the patient's clinical presentation with fever, joint pain, limited range of motion, and edematous eyelids, the etiology was more indicative of SLE than autoimmune encephalitis, which is typically isolated. Although the patient refused CSF studies, which would have confirmed the diagnosis, her symptoms did not meet the criteria for autoimmune encephalitis, as SLE could not be reasonably excluded as the primary cause [12].

**Table 1:** Laboratory studies and values on presentation (A) Metabolic panel and values, (B) hematology study and values, and (C) other laboratory studies and values/notes. (Abnormal values bolded.)

	Value/notes	Reference range
(A) Metabolic panel		
Sodium (mEq/L)	137	136–145
Potassium (mEq/L)	3.9	3.5–5.0
Chloride (mEq/L)	102	95–105
Bicarbonate (mEq/L)	22	23–26
Glucose (mg/dL)	120	70–99
BUN (mg/dL)	5	8–20
Creatinine (mg/dL)	0.42	0.8–1.3
Calcium (mg/dL)	8.3 (9.1 corrected for albumin)	9.0–10.5
Albumin (g/dL)	3.0	3.5–5.5
(B) Hematology study		
WBC count (10^3^/μl)	2.18	4–10
RBC count (10^6^/μl)	3.87	4.2–5.4
Hemoglobin (g/dL)	9.3	12–16
Hematocrit (%)	28.6	37–47
MCV (fL)	73.9	80–98
MCH (pq)	24	28–32
MCHC (q/dL)	32.5	33–36
RDW	17.2	9.0–14.5
PLT count (10^4^/dL)	380	150–350
ESR (mm/hr)	49	0–20
(C) Other laboratory studies		
Coagulation	Positive lupus anticoagulant	
Anti-cardiolipin IgG	Normal	
C-reactive protein (mg/dL)	1.7	<0.3
Blood culture/infectious panel	Normal	
Urinanalysis/urinary toxicology	Normal	

**Table 2:** Hospital course The hospital course from day 0 to day 7, showing vital signs, mental status, and SLE symptoms. Days 4-6 after an increase of steroid management showed improvement in both neuropsychiatric and systemic symptoms of SLE.

Day	Clinical summary
0	Aggressive, combative-haloperidol 5 mg IM × 1; lorazepam 1 mg IV × 2 given Febrile (*T*_max_ = 39, HR 130–140s); tylenol 1000 mg IV × 1; and Toradol 15 mg IV × 1 given immunosuppressed with pending sepsis workup; acyclovir 700 mg IV × 1, vancomycin 1000 mg IV × 1, ceftriaxone 2 g qD × 4 days initiated
1	Guarded and paranoid; code M called; 1:1 initiated - Olanzapine 5 mg PO QD given Febrile (*T*_max_ = 38.4). Mood described as tearful and remorseful that she has returned to the hospital "in handcuffs."
2	Delirious, irritable, with flat affect, patient refused lumbar puncture with capacity Febrile (*T*_max_ = 37.8), SLE worsening: increased joint pain albumin decreased to 2.7, from 3.2 CRP increased from 1.7 to 6.5
3	Improved mood; at baseline mental status per family Febrile (*T*_max_ 38.3) SLE worsening: joint pain/swelling, limited range of motion, edematous eyelids
4-6	Afebrile, good mood, feeling well overall
7	Afebrile, olanzapine helping with mood, cyclophosphamide therapy initiated

Discharge

On discharge from the hospital admission discussed above, the patient was grossly at her neuropsychiatric baseline with remission of psychotic symptomatology and, in terms of psychotropics, on olanzapine 5 mg nightly alone. She was also on her home hydroxychloroquine 400 mg daily in addition to related organ-sparing agents (e.g., lisinopril) and had a date-specified plan to complete her induction cyclophosphamide and rituximab infusion cycles via outpatient pediatric rheumatology follow-up. She completed her steroid taper prior to discharge.

Patient follow-up

She unfortunately did have another admission (albeit a brief one) to our hospital about one week after discharge, presenting at the time with acute psychosis. Etiology remained consistent with LC as the patient improved significantly with an additional steroid course and was likely exacerbated by psychotropic noncompliance as she had missed up to two doses during the preceding week in the community. There was some consideration about starting low-dose escitalopram due to concerns about an anxiety disorder, but this was ultimately deferred as any anxiety symptoms were instead determined to be due to an adjustment pathology related to her severe SLE. She was again discharged with the same medication regimen (and rheumatologic plan) as her prior discharge, in addition to an oral steroid taper.

She presented to our outpatient psychiatry department about one week after her second hospital discharge. Initial evaluation was consistent with a resolving psychotic process, with no evidence of thought disorganization yet notable guardedness, restricted range affect, and response latency on the mental status exam. The patient was adamant about continuing olanzapine only while completing her steroid taper due to a subjective belief that it was the cause of her psychiatric symptoms and that she did not want to be on anything that "affects mood." Anxiety symptoms continued to appear related to adjustment pathology, and the patient was given extensive psychotherapy resources to look for a therapist in the community.

On follow-up two to three weeks later, the patient appeared to be essentially at her neuropsychiatric baseline and expressed her own plan to discontinue olanzapine altogether, despite still being on a steroid taper. This was strongly advised against, and the patient was seen at two-week intervals for the next several appointments to ensure a lack of symptomatic relapse without any neuroleptic treatment. She remained asymptomatic, per both face-to-face evaluation and collateral obtained from her mother, and was functioning well, with good grades in her undergraduate studies. She was subsequently scheduled for appointments every four weeks and continued to be without any neuropsychiatric symptoms. Given her symptomatic stability and completion of her induction regimen through rheumatology, the patient ultimately requested discharge from our psychiatric practice; after discussion, she agreed to defer such until she establishes care with a psychotherapist near her academic institution and her current psychiatric provider is able to provide professional collateral to her new therapist.

## Discussion

The case in question highlights the importance of differentiating between a repeat steroid-induced psychotic episode and lupus cerebritis in a young female patient presenting with neuropsychiatric symptoms. Both conditions can manifest with similar behavioral changes and symptoms of psychosis, but understanding the patient's history, prior psychiatric history, family psychiatric history, medications, and associated risk factors is crucial for an accurate diagnosis. Distinguishing between these conditions is essential in emergency care settings because their treatments diverge significantly. SIP requires reducing steroid dosage, while LC necessitates aggressive immunosuppression and higher-dose steroids to control the inflammatory response.

In systemic lupus erythematosus, NP symptoms are relatively common, with around 52.1% of patients experiencing them within nine years of diagnosis [13]. These symptoms encompass a range of CNS and PNS issues, including headaches, cerebrovascular disease, mood disorders, anxiety, and seizures. Psychotic episodes, the rarest manifestation of NP events, are generally characterized by delusions with paranoia, along with reports of auditory, visual, and olfactory hallucinations, which lead to clinical distress and impairment in daily life [14]. Of the patients with SLE studied, only 1.53% experienced psychotic events, most often within three years of diagnosis [13]. Notable risk factors for NPSLE symptoms include young age at diagnosis, African ancestry, concurrent neuropsychiatric events, and the presence of anti-ribosomal P protein antibodies [13]. In this case, the patient had these risk factors. Her second psychotic episode within a six-month span was also noteworthy.

Lupus cerebritis is a rare manifestation of NPSLE. In longitudinal studies, the incidence of classified major CNS events correlated with high levels of inflammation and disease activity, some being seizures, strokes, myelopathy, optic neuritis, and psychosis, was noted to be only 7.8/100 person-years [4]. Laboratory analysis has narrowed down the finding that, specifically, anti-MOG antibodies and antiphospholipid antibodies are more involved with the CNS and associated with neuropsychiatric SLE [15]. While the patient did not experience any other acute CNS events and refused CSF antibody studies, her history of psychiatric symptoms, systemic symptoms, and MRI findings are most suggestive of LC.

Steroid-induced psychiatric conditions have a separate and distinct etiology and can vary depending on the dose and duration of corticosteroid administration. The exact mechanism is not fully understood, but exogenous corticosteroids can affect the hypothalamic-pituitary-adrenal (HPA) axis, leading to cognitive impairment and emotional disturbances [16,17]. Minor neuropsychiatric consequences include insomnia, mood swings, and personality changes, while severe consequences such as psychosis, mania, depression, and delirium can occur [16]. The risk of neuropsychiatric complications is higher with corticosteroid doses of 40 mg or more per day [18]. Initially, the patient's history of a prior steroid-induced psychotic episode and her dosage of steroids at the time suggested a potential repeat event on the differential, although MRI findings did not support a purely steroid-induced scenario.

The management of neuropsychiatric SLE involves investigating alternative causes, such as infection or malignancy, before addressing pure NPSLE. High-dose glucocorticoids and intravenous cyclophosphamide are the mainstays of treatment for severe symptoms resulting from inflammation or underlying autoimmune processes [19]. Other options include rituximab, intravenous immunoglobulins, or plasmapheresis, depending on the patient's response. Neurologic symptoms are treated with appropriate medications, including anti-epileptics for seizures, while psychiatric manifestations are targeted with anxiolytics, antidepressants, mood stabilizers, and/or antipsychotics, depending on the clinical presentation [20]. In the case of the patient in question, high-dose steroid therapy improved her psychiatric symptoms, supporting the diagnosis of a lupus flare with cerebritis.

For steroid-induced psychosis, the primary approach is to discontinue the offending steroid and, in some cases, use antipsychotic medications. Most patients recover from acute psychosis within an average of two weeks after treatment initiation [21]. In this case, antipsychotics were used in medical management, but steroids were increased, suggesting an underlying etiology more consistent with LC than SIP.

Ultimately, MRI is a crucial tool for assessing the brain in NPSLE. It can reveal subtle brain changes, including subcortical white matter lesions, cerebral atrophy, and other abnormalities [5,7,22]. Advanced MRI techniques, such as diffusion-tensor imaging and volumetric studies, provide additional information on brain microstructure and function in NPSLE [7]. In this case, timely MRI findings, which indicated subtle symmetric swelling with FLAIR hyperintensity and subtle DWI hyperintensity/ADV hypointensity representing restricted diffusion at the bilateral posterior limb of the internal capsule, caudate nuclei head, and parahippocampal gyri, supported the diagnosis of cerebral involvement related to lupus cerebritis. This ultimately aided in the patient's appropriate treatment and subsequent improvement of her neuropsychiatric symptoms on discharge.

Limitations

Limitations of this case include lack of access to outside hospital records, including detailed psychiatric history, and the patient declining further CSF studies with a lumbar puncture. A lumbar puncture with an antibody panel would have appropriately ruled out infectious etiology, autoimmune encephalitis, or definitively confirmed cerebritis.

## Conclusions

Patients with SLE who present with neuropsychiatric features need proper assessment to direct treatment plans. Lupus cerebritis can have similar presentations to steroid-induced psychosis. Taking into account a patient’s SLE, family, psychiatric, and medication history is vital to appropriate management. Notably, MRI imaging is critical management in differentiating SIP from LC and directing appropriate treatment, leading to improved patient outcomes.
